# Fractalkine receptor is expressed in mature ovarian teratomas and required for epidermal lineage differentiation

**DOI:** 10.1186/1757-2215-6-57

**Published:** 2013-08-17

**Authors:** Lisa Rooper, Hilal Gurler, Andre A Kajdacsy-Balla, Maria V Barbolina

**Affiliations:** 1 Departments of Pathology, University of Illinois at Chicago, 833 S Wood Str, Chicago, IL 60612, USA; 2 Biopharmaceutical Sciences, University of Illinois at Chicago, 833 S Wood Str, Chicago, IL 60612, USA

**Keywords:** Mature ovarian teratoma, Fractalkine receptor, Cell differentiation

## Abstract

**Background:**

The goal of this study was to determine a predominant cell type expressing fractalkine receptor (CX_3_CR1) in mature ovarian teratomas and to establish functional significance of its expression in cell differentiation.

**Methods:**

Specimens of ovarian teratoma and human fetal tissues were analyzed by immunohistochemistry for CX_3_CR1expression. Ovarian teratocarcinoma cell line PA-1 was used as a model for cell differentiation.

**Results:**

We found that the majority of the specimens contained CX_3_CR1-positive cells of epidermal lineage. Skin keratinocytes in fetal tissues were also CX_3_CR1- positive. PA-1 cells with downregulated CX_3_CR1 failed to express a skin keratinocyte marker cytokeratin 14 when cultured on Matrigel in the presence of a morphogen, bone morphogenic protein 4 (BMP-4), as compared to those expressing scrambled shRNA.

**Conclusions:**

Here we demonstrate that CX_3_CR1 is expressed in both normally (fetal skin) and abnormally (ovarian teratoma) differentiated keratinocytes and is required for cell differentiation into epidermal lineage.

## Background

Ovarian teratoma (dermoid cyst) is a benign tumor originating in germ cells. This tumor is one of the most common germ cell tumors and accounts for up to 20% of all ovarian cysts [1]. Ovarian teratoma is the most common tumor associated with pregnancy [2]. Generally, ovarian teratomas are cured by a complete surgical resection; however, some cases could give rise to a malignant disease [3,4]. Ovarian teratomas could be of both homozygous and heterozygous origin, as well as the mixture of the two, and it has been previously suggested that several mechanisms could lead to their development, including failure of meiosis I, failure of meiosis II, or duplication of a mature ovum [5]. Mature ovarian teratomas often contain a mixture of fully differentiated tissues from all three cell layers, ectodermal, endodermal, and mesodermal, resulting in formation of skin, hair follicle, sebaceous gland, bone, teeth, thyroid, and other cell types [6,7].

Our previous data obtained using a limited number of subjects suggested that a chemokine receptor fractalkine (CX_3_CR1) is expressed in ovarian teratomas [8]. CX_3_CR1 is a G protein-coupled receptor with multiple functions in both the normal state and disease [9,10]. CX_3_CR1 is activated by its highly specific ligand fractalkine (CX_3_CL1) [11]. It has been shown that CX_3_CL1/CX_3_CR1 axis plays a role in differentiation of osteoclasts [12] and development of dendritic cells [13], but its role in skin differentiation has not been described.

The goal of this study was to determine the predominant type of CX_3_CR1-positive cells in ovarian teratomas. We also aimed to determine whether the same cell type(s) are CX_3_CR1-positive during the normal development. Lastly, using a cell culture model, we intended to establish whether CX_3_CR1 expression is required for cell differentiation.

## Methods

### Materials

Tissue microarray (TMA) slides containing specimens of ovarian teratoma (Cat# OV805) and specimens of human fetal tissues (Cat# BE01014) were obtained from US Biomax (Rockville, MD). Because these specimens were commercially available and were deidentified, no approvals by the Institutional Review Board were required. GFP-tagged CX_3_CR1 shRNA and scrambled shRNA constructs were obtained from Origene Technologies (Rockville, MD). DharmaFECT was obtained from Dharmacon (Lafayette, CO). Matrigel was obtained from BD Biosciences (Bedford, MA). Mouse monoclonal CK14 and CK18 and rabbit polyclonal CX_3_CR1 (Cat# ab49747, ab49824, ab8021 and ab8020, respectively) antibodies were obtained from Abcam (Cambridge, MA), and mouse monoclonal β-tubulin antibody was obtained from Developmental Studies Hybridoma Bank (Iowa City, IA), goat polyclonal CX_3_CL1 (Cat# AF365) was purchased from R&D systems (Minneapolis, MN), and mouse monoclonal actin antibody was purchased from Santa Cruz Biotechnology (Dallas, TX). Vectastain ABC and DAB kits were obtained from Vector Laboratories (Burlingame, CA). BMP-4 was purchased from Invitrogen (Carlsbad, CA).

### Cell culture and directed differentiation

Ovarian teratocarcinoma cell line PA-1 and ovarian carcinoma cell line SKOV-3 were obtained from American Tissue Culture Collection (Manassas, VA). PA-1 cells were plated on Matrigel-coated (at 1/100 dilution) tissue culture plates or glass cover slips at 5% confluence and cultured in minimal essential media supplemented with sodium pyruvate and non-essential amino acids for 2 weeks. Cells subjected to differentiation into skin lineage were cultured in the above described media containing 10 μg/ml BMP-4.

### Immunohistochemical staining

TMA slides were deparaffinized by baking at 60°C for 2 h and rehydrated by incubation in xylene, 100% ethanol, 95% ethanol, 70% ethanol, and phosphate buffered saline, pH 7.4, for 5 min each. Peroxidase activity was inhibited with hydrogen peroxide. Antigen retrieval was performed by 15 min incubation in 1 mM ethylene diamine tetraacetic acid (EDTA; pH 8.0) at 95°C. Prior to primary antibody staining (1:50 dilution, rabbit anti-human CX_3_CR1, Abcam Cat# ab8021, 1 h at room temperature (RT)) non-specific binding was blocked by incubation with 10% goat serum for 1 h. The biotin-conjugated goat anti-rabbit secondary antibody was used at a 1:200 dilution for 30 min at RT. The Vectashield ABC kit was used as directed by the manufacturer, and tissues were incubated for 45 min at RT. The DAB reagent was prepared as instructed by the manufacturer and applied to tissues on TMA slides for 2 – 10 min until brown color developed. TMAs were stained with hematoxylin, dehydrated in 50%, 70%, 95%, and 100% ethanol, and mounted with Permount. Pancreatic cancer tissue was used as a positive control. Staining was evaluated by A.K.-B. and L.R., who were both blinded to the experimental outcomes of the study. Staining was scored based on the intensity and percentage of positive cells. Intensity of staining was “0” for negative samples, “1” for weakly positive samples, “2” for moderately positive samples, and “3” for highly positive samples. Overall scores were derived as the intensity score multiplied by the percentage. Staining was assessed separately in the cytoplasm and the membrane.

### Cell transfection and generation of stable cell lines

PA-1 cells were cultured to 80% confluence and transfected with either CX_3_CR1-specific GFP-tagged shRNA construct or scrambled GFP-tagged shRNA construct (Origene Technologies) using DharmaFECT according to the manufacturer’s instructions. Selection of the clones was performed with puromycin and green fluorescence as suggested by the manufacturer.

### Immunofluorescence staining

Cells were cultured on glass coverslips to 50-70% confluence, fixed, permeabilized with 0.01% Triton X-100, and blocked in goat serum. Mouse monoclonal CK-14 (Cat# ab49747) antibody was used at a dilution of 1:50. Cells were incubated with the primary antibodies for 1 h at room temperature (22°C). The cells were then incubated with secondary Alexa555-conjugated anti-mouse antibodies (1:500) for 1 h at RT in the dark. 4’,6-Diamidino-2-phenylindole (DAPI) was added to the secondary antibody solution to a final concentration of 10 μg/ml 10 min prior to the end of the incubation period. Cells were washed, air dried, and mounted on glass slides using ProlongGold (Invitrogen, Carlsbad, CA). Fluorescent imaging was performed using a Zeiss AxioObserverD.1 fluorescent microscope.

### Western blotting

Western blotting analysis was used to detect the expression of CK14, CK18, CX_3_CR1, CX_3_CL1, actin, and β-tubulin in PA-1. This procedure was performed as previously described [8,14,15] Antibodies were used at the following dilutions: 1:500 mouse anti-human-CK14 or CK18, or CX_3_CL1, rabbit anti-human CX_3_CR1 (Abcam ab8020) and 1:200 mouse anti-human-β-tubulin or goat anti-human actin in 3% BSA in a solution of 50 mM tris-buffered saline, pH 7.4, 150 mM NaCl, and 0.05% Tween-20 (TBST). Immunoreactive bands were visualized with anti-(mouse-IgG)-peroxidase, anti-(goat-IgG)-peroxidase, and anti-(rabbit-IgG)-peroxidase (Sigma, St. Louis, MO) (1:1000 in 3% BSA in TBST), and enhanced chemiluminescence was read using Chemidoc (Bio-Rad) and Bio-Rad Chemidoc ImageReader software.

## Results

### CX_3_CR1 is expressed in specimens of ovarian teratoma

**Figure 1:**
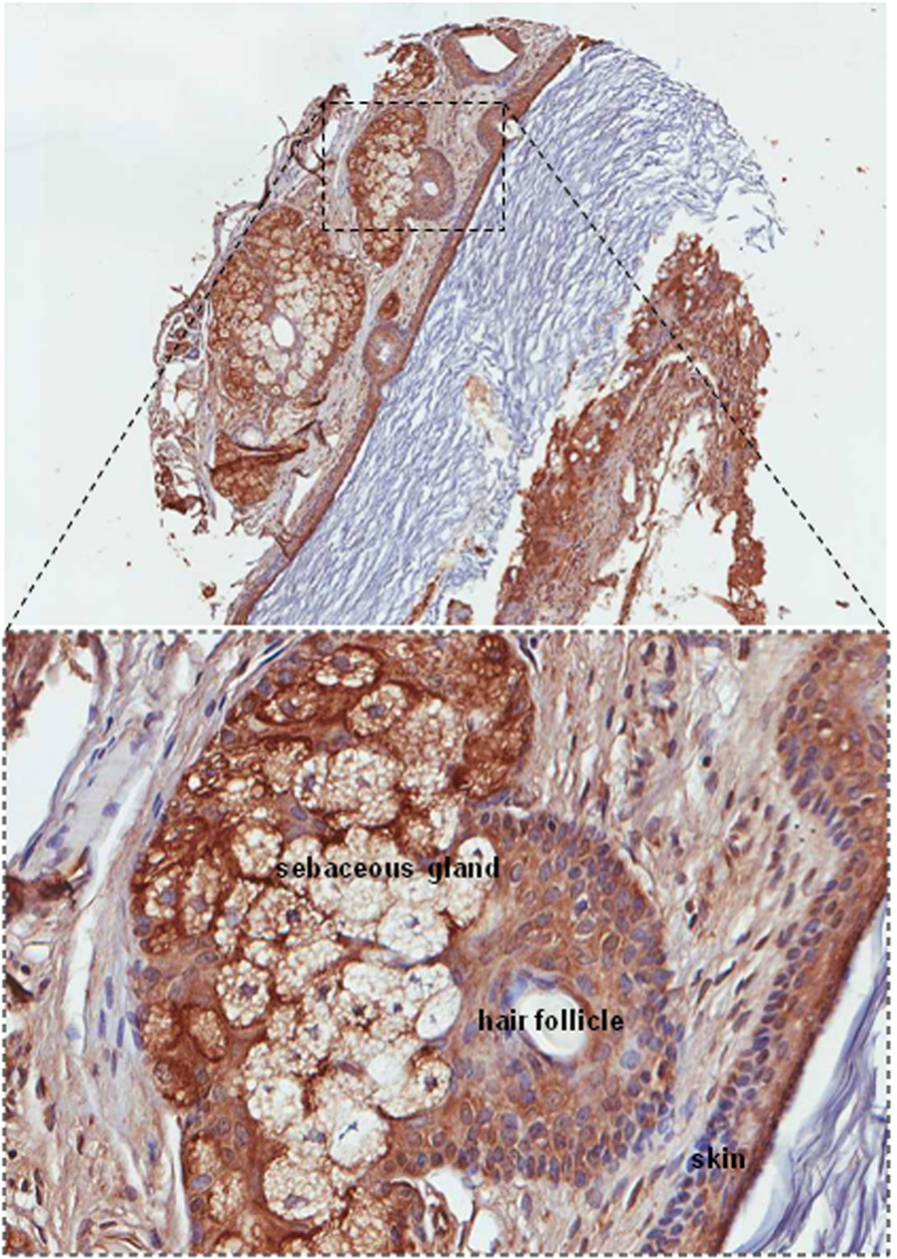
**CX**_**3**_**CR1 is expressed in human ovarian teratoma.** Specimens of ovarian teratoma were examined for CX_3_CR1 expression by immunohistochemistry. Brown – CX_3_CR1; blue – hematoxylin. Images were generated using an Aperio ScanScope digital slide scanner. Magnification – 5×. A region outlined by a dotted line is shown at 20× magnification. Cells of the sebaceous gland, hair follicle, and skin are indicated.

According to our previous observations CX_3_CR1 was expressed in cells comprising ovarian teratoma tissue specimens [8], although the number of specimens was very limited in order to be able to draw any conclusions. In the present study we aimed to expand this observation on a larger number of ovarian teratoma specimens and determine the predominant cell type(s) positive for CX_3_CR1. Thus, we tested expression of CX_3_CR1 using a tissue microarray containing 68 specimens of mature ovarian teratoma, 10 specimens of immature teratoma, and 2 cases of monodermal teratoma. Interestingly, we have found that skin keratinocytes represent the most predominant CX_3_CR1-positive cell type, which was present in 38% of tested cases of mature ovarian teratoma (Table 1, Figure 1). The next most common CX_3_CR1-positive types of cells in the tested cases of dermoid cyst were those comprising the pilosebaceous unit of the skin assembled of sebaceous gland and hair follicle (Table 1, Figure 1). Other CX_3_CR1-positive cell types in the order of diminution included lymphatic, apocrine, adipose, thyroid, respiratory epithelial, peripheral nervous, endothelial, and eccrine (Table 1). Cases of tested immature ovarian teratoma were characterized by the presence of CX_3_CR1-positive brain, lymphatic, respiratory epithelial, adipose, and sebaceous cells (Table 1). Two tested monodermal teratomas consisted of CX_3_CR1-positive thyroid and lymphatic tissues, respectively (Table 1). Thus, our data suggest that skin keratinocytes are the predominant type of CX_3_CR1-positive cells in the tested specimens of mature ovarian teratomas.

**Table 1:** Expression of CX3CR1 in human specimens of ovarian teratoma

**Position**	**Number**	**Sex**	**Age**	**Organ**	**Pathology**	**Grade**	**Type**	**Tissue type**	Intensity*	Location**	Percentage***
A1	1	F	28	Ovary	Mature teratoma	-	Benign	Sebaceous	2.5	C	100
A1	1	F	28	Ovary	Mature teratoma	-	Benign	Apocrine	1	C	10
A2	2	F	24	Ovary	Mature teratoma	-	Benign	Skin	2	C	20
A2	2	F	24	Ovary	Mature teratoma	-	Benign	Lymphocytes	3	N	90
A3	3	F	26	Ovary	Mature teratoma	-	Benign	NR			
A4	4	F	33	Ovary	Mature teratoma	-	Benign	Skin	3	C	100
A4	4	F	33	Ovary	Mature teratoma	-	Benign	Sebaceous	3	C	100
A5	5	F	48	Ovary	Mature teratoma	-	Benign	Follicle	3	C	100
A6	6	F	19	Ovary	Mature teratoma	-	Benign	Skin	3	C	100
A6	6	F	19	Ovary	Mature teratoma	-	Benign	Endothelium	1.5	C	90
A7	7	F	52	Ovary	Mature teratoma	-	Benign	Skin	2.5	C	100
A7	7	F	52	Ovary	Mature teratoma	-	Benign	Sebaceous	2.5	C	100
A8	8	F	27	Ovary	Mature teratoma	-	Benign	Skin	2	C	100
A8	8	F	27	Ovary	Mature teratoma	-	Benign	Apocrine	3	C	100
A8	8	F	27	Ovary	Mature teratoma	-	Benign	Lymph	3	NM	50
A9	9	F	22	Ovary	Mature teratoma	-	Benign	Sebaceous	3	C	100
A10	10	F	54	Ovary	Mature teratoma	-	Benign	Skin	2	C	100
A10	10	F	54	Ovary	Mature teratoma	-	Benign	Sebaceous	2.5	C	100
B1	11	F	58	Ovary	Mature teratoma	-	Benign	Adipose	1	C	80
B1	11	F	58	Ovary	Mature teratoma	-	Benign	Adipose	3	M	40
B2	12	F	32	Ovary	Mature teratoma	-	Benign	Skin	2	C	100
B2	12	F	32	Ovary	Mature teratoma	-	Benign	Follicle	2.5	C	100
B2	12	F	32	Ovary	Mature teratoma	-	Benign	Sebaceous	3	C	100
B3	13	F	28	Ovary	Mature teratoma	-	Benign	NR			
B4	14	F	40	Ovary	Mature teratoma	-	Benign	Adipose	1	C	60
B4	14	F	40	Ovary	Mature teratoma	-	Benign	Adipose	3	M	40
B5	15	F	26	Ovary	Mature teratoma	-	Benign	Sebaceous	3	C	70
B5	15	F	26	Ovary	Mature teratoma	-	Benign	Apocrine	3	C	100
B6	16	F	39	Ovary	Mature teratoma	-	Benign	Endothelium	3	C	100
B6	16	F	39	Ovary	Mature teratoma	-	Benign	Lymphocytes	3	N	70
B7	17	F	23	Ovary	Mature teratoma	-	Benign	Lymphocytes	3	N	40
B7	17	F	23	Ovary	Mature teratoma	-	Benign	Apocrine	2.5	C	30
B8	18	F	32	Ovary	Mature teratoma	-	Benign	Sebaceous	3	C	100
B8	18	F	32	Ovary	Mature teratoma	-	Benign	Skin	3	C	100
B8	18	F	32	Ovary	Mature teratoma	-	Benign	Thyroid	3	C	80
B9	19	F	29	Ovary	Mature teratoma	-	Benign	Thyroid	3	C	80
B10	20	F	30	Ovary	Mature teratoma	-	Benign	Follicle	2	C	100
C1	21	F	40	Ovary	Mature teratoma	-	Benign	Sebaceous	3	C	100
C1	21	F	40	Ovary	Mature teratoma	-	Benign	Skin	1.5	C	100
C2	22	F	35	Ovary	Mature teratoma	-	Benign	Sebaceous	3	C	100
C2	22	F	35	Ovary	Mature teratoma	-	Benign	Skin	3	C	100
C3	23	F	49	Ovary	Mature teratoma	-	Benign	Lymphocytes	3	N	10
C4	24	F	51	Ovary	Mature teratoma	-	Benign	Skin	3	C	100
C5	25	F	22	Ovary	Mature teratoma	-	Benign	Skin	2	C	100
C5	25	F	22	Ovary	Mature teratoma	-	Benign	Lymphocytes	2	N	10
C6	26	F	69	Ovary	Mature teratoma	-	Benign	NR			
C7	27	F	35	Ovary	Mature teratoma	-	Benign	Sebaceous	3	C	100
C7	27	F	35	Ovary	Mature teratoma	-	Benign	Follicle	2	C	100
C7	27	F	35	Ovary	Mature teratoma	-	Benign	Apocrine	2.5	C	90
C8	28	F	8	Ovary	Mature teratoma	-	Benign	Apocrine	2.5	M	100
C8	28	F	8	Ovary	Mature teratoma	-	Benign	Apocrine	1	C	50
C9	29	F	23	Ovary	Mature teratoma	-	Benign	Sebaceous	3	C	100
C9	29	F	23	Ovary	Mature teratoma	-	Benign	Skin	3	C	90
C10	30	F	26	Ovary	Mature teratoma	-	Benign	NR			
D1	31	F	35	Ovary	Mature teratoma	-	Benign	Sebaceous	3	C	100
D1	31	F	35	Ovary	Mature teratoma	-	Benign	Apocrine	3	C	60
D2	32	F	47	Ovary	Mature teratoma	-	Benign	Skin	2	C	100
D2	32	F	47	Ovary	Mature teratoma	-	Benign	Sebaceous	3	C	100
D3	33	F	56	Ovary	Mature teratoma	-	Benign	Adipose	1	C	60
D3	33	F	56	Ovary	Mature teratoma	-	Benign	Adipose	3	M	40
D3	33	F	56	Ovary	Mature teratoma	-	Benign	Apocrine	3	C	70
D4	34	F	40	Ovary	Mature teratoma	-	Benign	NR			
D5	35	F	32	Ovary	Mature teratoma	-	Benign	Skin	3	C	100
D5	35	F	32	Ovary	Mature teratoma	-	Benign	Sebaceous	3	C	100
D6	36	F	12	Ovary	Mature teratoma	-	Benign	Lymphocyte	3	N	40
D6	36	F	12	Ovary	Mature teratoma	-	Benign	Apocrine	3	C	70
D7	37	F	43	Ovary	Mature teratoma	-	Benign	NR			
D8	38	F	27	Ovary	Mature teratoma	-	Benign	Skin	2	C	100
D9	39	F	22	Ovary	Mature teratoma	-	Benign	Sebaceous	3	C	100
D9	39	F	22	Ovary	Mature teratoma	-	Benign	Eccrine	1	C	30
D10	40	F	40	Ovary	Mature teratoma	-	Benign	Adipose	1	C	60
D10	40	F	40	Ovary	Mature teratoma	-	Benign	Adipose	3	M	30
D10	40	F	40	Ovary	Mature teratoma	-	Benign	Skin	3	C	100
E1	41	F	52	Ovary	Mature teratoma	-	Benign	Respiratory epithelium	2.5	C	80
E2	42	F	23	Ovary	Mature teratoma	-	Benign	Sebaceous	3	C	100
E2	42	F	23	Ovary	Mature teratoma	-	Benign	Thyroid	1.5	C	70
E2	42	F	23	Ovary	Mature teratoma	-	Benign	Apocrine	2	C	70
E3	43	F	23	Ovary	Mature teratoma	-	Benign	Respiratory epithelium	2	C	50
E3	43	F	23	Ovary	Mature teratoma	-	Benign	Lymphocytes	3	N	20
E4	44	F	47	Ovary	Mature teratoma	-	Benign	Skin	3	C	100
E4	44	F	47	Ovary	Mature teratoma	-	Benign	Nerve	3	NM	80
E4	44	F	47	Ovary	Mature teratoma	-	Benign	Nerve	2	C	70
E5	45	F	45	Ovary	Mature teratoma	-	Benign	Skin	3	C	100
E6	46	F	33	Ovary	Mature teratoma	-	Benign	Sebaceous	3	C	100
E6	46	F	33	Ovary	Mature teratoma	-	Benign	Respiratory epithelium	1.5	C	100
E6	46	F	33	Ovary	Mature teratoma	-	Benign	Apocrine	2	C	70
E6	46	F	33	Ovary	Mature teratoma	-	Benign	Lymphocytes	3	N	5
E7	47	F	27	Ovary	Mature teratoma	-	Benign	Sebaceous	2.5	C	100
E7	47	F	27	Ovary	Mature teratoma	-	Benign	Lymphocytes	3	N	30
E7	47	F	27	Ovary	Mature teratoma	-	Benign	Skin	2	C	90
E8	48	F	28	Ovary	Mature teratoma	-	Benign	Adipose	1	C	100
E8	48	F	28	Ovary	Mature teratoma	-	Benign	Adipose	3	M	50
E8	48	F	28	Ovary	Mature teratoma	-	Benign	Follicle	3	C	90
E9	49	F	24	Ovary	Mature teratoma	-	Benign	NR			
E10	50	F	22	Ovary	Mature teratoma	-	Benign	Skin	3	C	100
F1	51	F	27	Ovary	Mature teratoma (ovary tissue)	-	Benign	NR			
F2	52	F	32	Ovary	Mature teratoma	-	Benign	Lymphocytes	3	N	100
F3	53	F	42	Ovary	Mature teratoma	-	Benign	NR			
F4	54	F	30	Ovary	Mature teratoma	-	Benign	Adipose	1	C	30
F4	54	F	30	Ovary	Mature teratoma	-	Benign	Adipose	3	M	15
F5	55	F	38	Ovary	Mature teratoma	-	Benign	Sebaceous	3	C	100
F5	55	F	38	Ovary	Mature teratoma	-	Benign	Skin	2.5	C	90
F5	55	F	38	Ovary	Mature teratoma	-	Benign	Lymphocytes	3	N	10
F6	56	F	36	Ovary	Mature teratoma	-	Benign	Sebaceous	3	C	100
F6	56	F	36	Ovary	Mature teratoma	-	Benign	Skin	3	C	100
F7	57	F	25	Ovary	Mature teratoma	-	Benign	Skin	3	C	100
F7	57	F	25	Ovary	Mature teratoma	-	Benign	Lymphocytes	3	N	90
F8	58	F	39	Ovary	Mature teratoma	-	Benign	Skin	3	C	100
F8	58	F	39	Ovary	Mature teratoma	-	Benign	Sebaceous	3	C	100
F9	59	F	32	Ovary	Mature teratoma	-	Benign	Sebaceous	3	C	100
F9	59	F	32	Ovary	Mature teratoma	-	Benign	Skin	3	C	100
F9	59	F	32	Ovary	Mature teratoma	-	Benign	Thyroid	2.5	C	50
F10	60	F	29	Ovary	Mature teratoma	-	Benign	Nerve	3	NM	100
F10	60	F	29	Ovary	Mature teratoma	-	Benign	Nerve	2.5	C	100
F10	60	F	29	Ovary	Mature teratoma	-	Benign	Adipose	1	C	10
F10	60	F	29	Ovary	Mature teratoma	-	Benign	Adipose	3	M	10
F10	60	F	29	Ovary	Mature teratoma	-	Benign	Follicle	2	C	100
F10	60	F	29	Ovary	Mature teratoma	-	Benign	Sebaceous	3	C	100
G1	61	F	32	Ovary	Mature teratoma	-	Benign	NR			
G2	62	F	36	Ovary	Mature teratoma	-	Benign	Adipose	1	C	20
G2	62	F	36	Ovary	Mature teratoma	-	Benign	Adipose	3	M	10
G3	63	F	29	Ovary	Mature teratoma	-	Benign	NR			
G4	64	F	39	Ovary	Mature teratoma	-	Benign	Nerve	3	NM	10
G5	65	F	15	Ovary	Immature teratoma	II	Malignant	Lymphocytes	3	N	10
G6	66	F	40	Ovary	Immature teratoma	-	Malignant	NR			
G7	67	F	29	Ovary	Immature teratoma	-	Malignant	Immature	3	C/N	90
G8	68	F	37	Ovary	Immature teratoma	-	Malignant	Immature	2.5	C	100
G9	69	F	39	Ovary	Mature teratoma	-	Benign	Skin	3	C	100
G10	70	F	40	Ovary	Mature teratoma	-	Benign	Sebaceous	3	C	100
G10	70	F	40	Ovary	Mature teratoma	-	Benign	Skin	3	C	100
H1	71	F	49	Ovary	Mature teratoma	-	Benign	Adipose	1	C	30
H1	71	F	49	Ovary	Mature teratoma	-	Benign	Adipose	3	M	10
H2	72	F	28	Ovary	Immature teratoma	II	Malignant	NR			
H3	73	F	37	Ovary	Immature teratoma	-	Malignant	Immature	1	C	70
H4	74	F	22	Ovary	Immature teratoma	II	Malignant	Respiratory epithelium	2	C	90
H4	74	F	22	Ovary	Immature teratoma	II	Malignant	Lymphocyte	3	N	30
H5	75	F	28	Ovary	Immature teratoma	II	Malignant	Adipose	1	C	100
H5	75	F	28	Ovary	Immature teratoma	II	Malignant	Respiratory epithelium	2.5	C	100
H5	75	F	28	Ovary	Immature teratoma	II	Malignant	Sebaceous	1	C	70
H6	76	F	20	Ovary	Immature teratoma	II	Malignant	Immature	1.5	C	70
H7	77	F	18	Ovary	Immature teratoma	III	Malignant	Cystic epithelium	1.5	C	90
H8	78	M	45	Ovary	Mature teratoma	-	Benign	Sebaceous	2.5	C	100
H8	78	M	45	Ovary	Mature teratoma	-	Benign	Lymphocytes	3	N	50
H9	79	F	47	Ovary	Malignant teratoma	-	Benign	Apocrine	1.5	C	50
H10	80	F	69	Ovary	Monodermal teratoma	-	Malignant	Thyroid	2.5	C	40
H10	80	F	69	Ovary	Monodermal teratoma	-	Malignant	Lymphocytes	3	N	30

* Intensity was graded in the scale from 0 to 3; 0 - no staining, 1 - weak staining, 2 - moderate staining, 3 - strong staining.

** Location of the CX_3_CR1 staining was cytoplasmic (C), nuclear (N), and membranous (M), as indicated.

*** Percentage of CX_3_CR1-positive cells is indicated.

### CX_3_CR1 is expressed in fetal tissues

Further we wished to determine CX_3_CR1-positive cells undergoing the normal development. We tested both male and female fetal tissues aged 5 months *in utero*. We found that the majority of epithelial cells, including skin keratinocytes, were CX_3_CR1-positive (Table 2). Hence, CX_3_CR1-positive skin cells are present not only within abnormally differentiated tissues, such as mature ovarian teratomas, but also in the normally developing skin.

**Table 2:** Expression of CX3CR1 in human fetal tissues

**Position**	**Number**	Sex*	Age*	Organ*	Type*	Intensity**	**Location**	Percentage***	Score****	**Notes**
A1	1	M	5 mon.	Heart	Normal	2.5		100	250	
A2	2	M	5 mon.	Heart	Normal	2.5		100	250	
A3	3	M	5 mon.	Heart	Normal	2.5		100	250	
A4	4	M	5 mon.	Gallbladder	Normal	2		100	200	
A5	5	M	5 mon.	Gallbladder	Normal	2		60	120	
A6	6	M	5 mon.	Gallbladder	Normal	2		90	180	
A7	7	M	5 mon.	Colon	Normal	3		100	300	
A8	8	M	5 mon.	Colon	Normal	3		100	300	
A9	9	M	5 mon.	Colon	Normal	3		100	300	
B1	10	M	5 mon.	Small intestine	Normal	2.5		100	250	
B2	11	M	5 mon.	Small intestine	Normal	2.5		100	250	
B3	12	M	5 mon.	Small intestine	Normal	2.5		100	250	
B4	13	M	5 mon.	Liver	Normal	2.5		100	250	
B5	14	M	5 mon.	Liver	Normal	3		100	300	
B6	15	M	5 mon.	Liver	Normal	3		100	300	
B7	16	M	5 mon.	Rectum	Normal	2.5		95	237.5	
B8	17	M	5 mon.	Rectum	Normal	2		95	190	
B9	18	M	5 mon.	Rectum	Normal	2.5		100	250	
C1	19	M	5 mon.	Stomach	Normal	2		70	140	
C2	20	M	5 mon.	Stomach	Normal	1.5		60	90	
C3	21	M	5 mon.	Stomach	Normal	1.5		80	120	
C4	22	M	5 mon.	Adrenal gland	Normal	2.5		80	200	
C5	23	M	5 mon.	Adrenal gland	Normal	2		90	180	
C6	24	M	5 mon.	Adrenal gland	Normal	2		60	120	
C7	25	M	5 mon.	Thyroid gland	Normal	0.5		30	15	
C8	26	M	5 mon.	Thyroid gland	Normal	0.5		10	5	
C9	27	M	5 mon.	Thyroid gland	Normal	0.5		10	5	
D1	28	M	5 mon.	Spleen	Normal	1		80	80	
D2	29	M	5 mon.	Spleen	Normal	1		80	80	
D3	30	M	5 mon.	Spleen	Normal	1		80	80	
D4	31	M	5 mon.	Thymus gland	Normal	2.5	Epithelioid cells	20	50	
D4						1	Lymphocytes	100	100	
D5	32	M	5 mon.	Thymus gland	Normal	2.5	Epithelioid cells	20	50	
D5						1	Lymphocytes	100	100	
D6	33	M	5 mon.	Thymus gland	Normal	2.5	Epithelioid cells	20	50	
D6						1	Lymphocytes	100	100	
D7	34	M	5 mon.	Skin	Normal	2	Epithelium	80	160	
D7						3	Adipose	70	210	
D8	35	M	5 mon.	Skin	Normal	1.5	Epithelium	80	120	
D8						3	Adipose	70	210	
D9	36	M	5 mon.	Skin	Normal	2	Epithelium	90	180	
D9						3	Adipose	80	240	
E1	37	M	5 mon.	Bone	Normal	2.5		30	75	
E2	38	M	5 mon.	Bone	Normal	2.5		30	75	
E3	39	M	5 mon.	Bone	Normal	2.5		30	75	
E4	40	M	5 mon.	Epididymis	Normal	1		90	90	
E5	41	M	5 mon.	Epididymis	Normal	0.5		60	30	
E6	42	M	5 mon.	Epididymis	Normal	0.5		70	35	
E7	43	M	5 mon.	Brain	Normal	2		20	40	
E8	44	M	5 mon.	Brain	Normal	1		5	5	
E9	45	M	5 mon.	Brain	Normal	1		5	5	
F1	46	M	5 mon.	Lung and trachea	Normal	3	Trachea	90	270	
F1	46	M	5 mon.	Lung and trachea	Normal	3	Alveoli	25	75	
F2	47	M	5 mon.	Lung and trachea	Normal	3	Trachea	90	270	
F2	47	M	5 mon.	Lung and trachea	Normal	1	Alveoli	25	25	
F3	48	M	5 mon.	Lung and trachea	Normal	3	Trachea	80	240	
F3	48	M	5 mon.	Lung and trachea	Normal	1	Alveoli	25	25	
F4	49	M	5 mon.	Muscle	Normal	0.5		100	50	
F5	50	M	5 mon.	Muscle	Normal	0.5		100	50	
F6	51	M	5 mon.	Muscle	Normal	0.5		100	50	
F7	52	M	5 mon.	Smooth muscle	Normal	2		100	200	
F8	53	M	5 mon.	Smooth muscle	Normal	2		100	200	
F9	54	M	5 mon.	Smooth muscle	Normal	2		100	200	
G1	55	M	5 mon.	Kidney	Normal	2	Tubules	80	160	
G1	55	M	5 mon.	Kidney	Normal	1	Glomeruli	50	50	
G2	56	M	5 mon.	Kidney	Normal	2.5	Tubules	70	175	
G2	56	M	5 mon.	Kidney	Normal	0.5	Glomeruli	30	15	
G3	57	M	5 mon.	Kidney	Normal	1.5	Tubules	90	135	
G3	57	M	5 mon.	Kidney	Normal	0.5	Glomeruli	70	35	
G4	58	M	5 mon.	Eye	Normal	3		100	300	Retinal pigment epithelium
G5	59	M	5 mon.	Eye	Normal	3		100	300	Retinal pigment epithelium
G6	60	M	5 mon.	Eye	Normal	3		100	300	Retinal pigment epithelium
G7	61	F	5 mon.	Umbilical cord	Normal	2		100	200	
G8	62	F	5 mon.	Umbilical cord	Normal	1.5		100	150	
G9	63	F	5 mon.	Umbilical cord	Normal	NA		NA	NA	
H1	64	F	5 mon.	Placenta	Normal	0.5		20	10	
H2	65	F	5 mon.	Placenta	Normal	0.5		10	5	
H3	66	F	5 mon.	Placenta	Normal	0.5		40	20	
H4	67	F	5 mon.	Uterus	Normal	0.5		20	10	
H5	68	F	5 mon.	Uterus	Normal	0.5		5	2.5	
H6	69	F	5 mon.	Uterus	Normal	0.5		20	10	
H7	70	F	5 mon.	Pancreas	Normal	2	Acini and ducts	80	160	
H8	71	F	5 mon.	Pancreas	Normal	2.5	Acini and ducts	80	200	
H9	72	F	5 mon.	Pancreas	Normal	2.5	Acini and ducts	70	175	
I1	73	F	5 mon.	Ovary	Normal	3		70	210	
I2	74	F	5 mon.	Ovary	Normal	3		80	240	
I3	75	F	5 mon.	Ovary	Normal	3		90	270	
I4	76	F	5 mon.	Fallopian tube	Normal	0.5		5	2.5	
I5	77	F	5 mon.	Fallopian tube	Normal	1		10	10	
I6	78	F	5 mon.	Fallopian tube	Normal	1.5		5	7.5	

* Information obtained from the manufacturer (US Biomax; Rockville, MD) for the multiple organ fetal tissue array BE01014; TMA contained triplicate cores (26cases/78cores).

** Intensity of staining was scored as negative (0), weak positive (1), moderate positive (2), and strong positive (3).

*** Denotes percentage of positive cells in the core.

**** Score has been calculated as intensity multiplied by percentage of positive cells.

### CX_3_CR1 is required for keratinocyte differentiation

**Figure 2:**
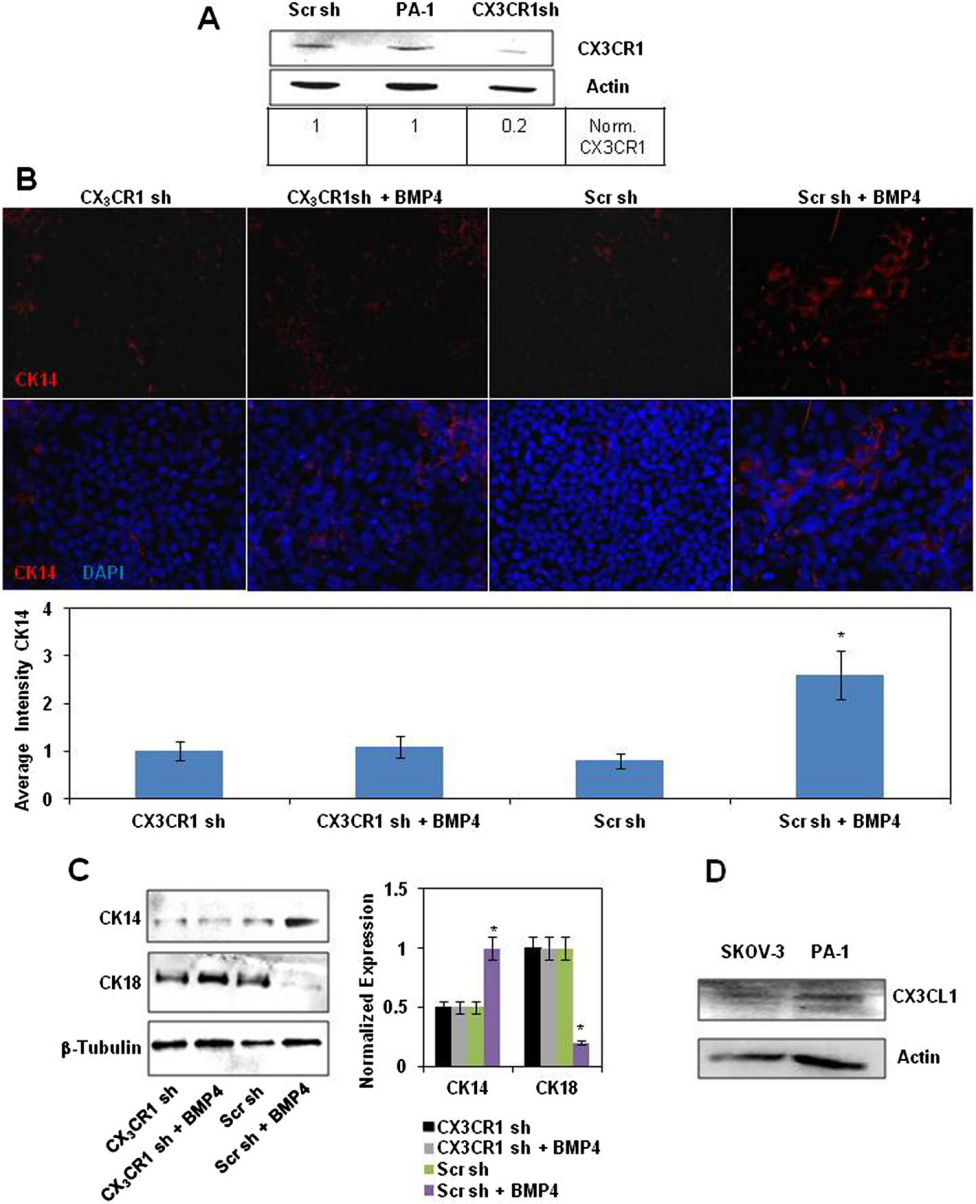
**CX**_**3**_**CR1 is required for keratinocytes differentiation. (A)** Expression of CX_3_CR1 in parental PA-1 cell line and that stably transfected with either CX_3_CR1 or scrambled shRNA constructs was examined with Western blot. Actin served as a loading control. CX_3_CR1 expression levels were quantified using digital densitometry and normalized to the levels of actin expression. **(B)** Expression of cytokeratin 14 in PA-1 cells stably transfected with either CX_3_CR1 or scrambled shRNA constructs treated with recombinant BMP-4 or vehicle, as indicated, was examined with immunocytochemistry. CK14 in PA-1 was probed with anti-CK14 and Alexa555-conjugated anti-rabbit antibodies, and DNA was stained with 4’,6-Diamidino-2-Phenylindole, Dihydrochloride (DAPI); CK14 – red, DNA – blue. Images were taken using 5× magnification on the objective. The histogram demonstrates the average intensity of CK14 signal across the field as determined by the Zeiss AxioVision software. Data is an average of three independent experiments. *p < 0.05. **(C)** Expression of cytokeratins 14 and 18 in PA-1 cells stably transfected with either CX_3_CR1 or scrambled shRNA constructs treated with recombinant BMP-4 or vehicle, as indicated, was examined with Western blot. β-Tubulin served as a loading control. The histogram shows CK14 and CK18 expression levels. Expression of CK18 in CX_3_CR1shRNA-transfected PA-1 cells was arbitrarily set as 1 and expression of both CK14 and CK18 in other conditions was calculated accordingly. The data represent a typical Western blot image, and quantitative analysis was performed using three independently performed experiments. *p < 0.05. **(D)** Expression of CX_3_CL1 in SKOV-3 and PA-1 cell lines was examined with Western blot. Actin served as a loading control. SKOV-3 cell line was used as a positive control.

Based on our observations regarding CX_3_CR1 expression in both normally and abnormally developing keratinocytes, we hypothesized that CX_3_CR1 may be important for cell differentiation into the epidermal lineage. Thus, we tested the requirement for CX_3_CR1 in keratinocyte differentiation using a cell culture model. Ovarian teratocarcinoma cell line PA-1 has been established from the ascites of a patient with malignant ovarian teratoma [16]. Chromosomal analysis had suggested that these cells have a heterozygous origin and derived at a stage prior to the first meiotic division [17]. Several studies have shown that PA-1 is capable of differentiation into diverse cell lineages at the appropriate culture conditions [16,18,19]. Based on many similarities between the teratocarcinoma cells and early embryonic cells, the former has been used to study processes of differentiation [19,20]. Using gene specific shRNA constructs, we downregulated CX_3_CR1 in PA-1 cell line (Figure 2A). Further we cultured both CX_3_CR1sh-PA-1 and the control cells transfected with a scrambled shRNA construct on Matrigel in the presence and absence of 0.5 nM BMP-4 for 2 weeks. Expression of cytokeratin 14 (CK14) and cytokeratin 18 (CK18) was analysed with immunofluorescence microscopy and Western blotting (Figure 2B,C). We observed that only PA-1 transfected with scrambled shRNA and cultured in BMP-4-supplemented media expressed significantly higher levels of CK14 compared to scrambled shRNA PA-1 or CX_3_CR1 shRNA PA-1cultured in the absence of BMP-4, as well as CX_3_CR1 shRNA PA-1 cultured in the presence of BMP-4 (Figure 2B,C). In opposite, scrambled shRNA PA-1 or CX_3_CR1 shRNA PA-1cultured in the absence of BMP-4, as well as CX_3_CR1 shRNA PA-1 cultured in the presence of BMP-4 expressed higher levels of CK18 compared to scrambled shRNA PA-1 cultured in the presence of BMP-4 (Figure 2C). PA-1 expressed fractalkine ligand (CX_3_CL1) necessary for the receptor (CX_3_CR1) activation (Figure 2D). These data suggest that CX_3_CR1-positive PA-1 cells are able to proceed toward differentiation into skin keratinocytes in the presence of BMP-4 and begin expressing a marker of stratified epithelium CK14, while the loss of CX_3_CR1 restricts differentiation and the cells remain CK18-positive simple-epithelial cells.

## Discussion

Our findings demonstrate that cells of the skin lineage comprise the most predominant CX_3_CR1-positive cell type within the specimens of ovarian teratoma. Mature skin is a predominant tissue found in mature ovarian teratomas [21]. Our findings also show that normally developed keratinocytes are CX_3_CR1-positive. Importantly, our findings suggest that CX_3_CR1 could be an important player in keratinocyte differentiation. Our data indicate that teratocarcinoma cell line PA-1 expressed a basal keratinocyte marker CK14 when cultured on Matrigel in the presence of BMP-4. In opposite, cells with silenced CX_3_CR1 and those cultured in the absence of BMP-4 expressed CK18 specific for simple non-stratified epithelium.

The role for CX_3_CR1 in skin differentiation has not been described before, although, the role of this receptor in skin wound healing has been reported [22]. Our findings could be potentially important, as skin is the largest organ of the human body. Many pathological conditions and injuries require regeneration of the skin, and affecting the CX_3_CR1-dependent pathway could be one way to achieving faster skin formation from cells capable of differentiation. Thus, the studies of the role of CX_3_CR1 in keratinocyte differentiation need to be expanded further to include more extensive studies on other abnormally differentiating teratoma cell lines, as well as normally differentiating embryonic stem cells. Furthermore, we observed that CX3CR1 is expressed in other developing tissues, as indicated in Table 2, thus, it is possible that this receptor could be important for the development of those tissues as well. In fact, a role for the fractalkine axis in osteoclasts differentiation has been reported, whereby CX_3_CL1-expressing osteoblasts regulate differentiation of CX_3_CR1-positive osteoclast precursors [12]. Hence, more rigorous future studies of the role of CX_3_CR1 in development and differentiation are required.

## Conclusions

Here we present that fractalkine receptor is expressed by skin cells in specimens of human ovarian teratoma and fetus and is required for epidermal lineage differentiation. This information sheds light on the ovarian pathology, dermoid cyst, and outlines possible mechanisms of cell differentiation within this benign formation. As the fractalkine receptor has recently emerged as a novel potential therapeutic target against many debilitating diseases, it is important to remember its role in cell differentiation when applying future anti-fractalkine therapies.

## Abbreviations

BMP-4: Bone morphogenic protein 4; BSA: Bovine serum albumin; CK14: Cytokeratin 14; CK18: Cytokeratin 18; CX3CL1: Fractalkine (chemokine ligand); CX3CR1: Fractalkine receptor (chemokine receptor); DAPI: 4’,6-diamidino-2-phenylindole; EDTA: Ethylenediaminetetraacetic acid; GFP: Green fluorescent protein; PA-1: Ovarian teratocarcinoma cell line; TMA: Tissue microarray.

## Competing interests

All authors declare neither financial nor non-financial competing interests.

## Authors’ contributions

LR analysed CX_3_CR1 expression in tissue microarrays and interpreted the data. HG analysed expression of CX_3_CR1 and CX_3_CL1. AAKB analysed CX_3_CR1 expression in tissue microarrays, interpreted the data, and contributed to study design. MVB conceived of the study, performed immunohistochemical staining, created stable clones with shRNA, performed immunofluorescence and Western blot experiments, and drafted the manuscript. All authors read and approved the final manuscript.

## Authors’ information

AAKB is a pathologist with specialization in gynecologic oncology. MVB is a basic cancer researcher whose main research interests are on metastatic ovarian carcinoma. Both AAKB and MVB share research interests on the role of CX_3_CL1/CX_3_CR1 axis in prostate and ovarian cancers, respectively. LR, AAKB, and MVB have recently co-authored an article describing a role of CX_3_CR1 in progression of metastatic ovarian carcinoma. This current study seeks to expand the understanding of the biological impact of the fractalkine axis on cell differentiation.

Study design: fractalkine receptor is expressed by skin cells in specimens of human ovarian teratoma and fetus and is required for epidermal lineage differentiation.
